# African-American Women’s Early-Life Exposure to Neighborhood Mortgage Discrimination and Preterm Birth Rates: A Population-Based Study

**DOI:** 10.1007/s10995-025-04171-x

**Published:** 2025-09-19

**Authors:** Christina Kim, S. J. Cavé Doi, Liz Lamere, Kristin Rankin, Nana Matoba, Nikhil Prachand, James W. Collins

**Affiliations:** 1 Ann & Robert H. Lurie Children’s Hospital, McGaw Medical Center of Northwestern University, Chicago, IL USA; 2 Chicago Department of Public Health, Chicago, IL USA; 3 University of California San Diego, San Diego, CA USA; 4 University of Illinois Chicago School of Public Health, Chicago, IL USA

**Keywords:** Mortgage discrimination, Structural racism, Preterm birth, Life-course model

## Abstract

**Objective:**

To determine the extent to which African-American women’s early-life residence in urban neighborhoods with mortgage discrimination (compared to neighborhoods without mortgage discrimination) is associated with preterm birth (< 37 weeks, PTB).

**Methods:**

Stratified and multivariable binominal regression analyses were performed on a Chicago transgenerational dataset of African-American women (born 1989–1991) and their infants (born 2005–2017) with appended Home Mortgage Disclosure Act and Index of Concentration at the Extremes (ICE) data.

**Results:**

In mortgage discriminated neighborhoods, the proportion of non-Hispanic White residents exceeded that of neighborhoods without mortgage discrimination: 84% vs. 31%, *p* < 0.01. Additionally, mean ICE_race/ethnicity_ for mortgage discriminated neighborhoods equaled 0.78 (0.64–0.91) confirming the greater concentrations of non-Hispanic White populations. African-American women (*n* = 735) with early-life residence in mortgage discriminated neighborhoods had a PTB rate of 15.8% compared to 13.1% for those (*n* = 23,369) with early-life residence in non-mortgage discriminated neighborhoods; RR = 1.20 (1.01, 1.43). The adjusted (controlling for trimester of prenatal care usage and cigarette smoking) RR of early (< 34 weeks), late (34–36 weeks), and total PTB for African-American women with early-life residence in mortgage (compared to non-mortgage discriminated) neighborhoods equaled 1.60 (1.20, 2.14), 1.18 (0.92,1.53), and 1.31 (1.09,1.57), respectively. The subgroup of African-American women (*n* = 536) with early-life residence in mortgage discriminated neighborhoods and adulthood residence in non-mortgage discriminated neighborhoods had an early PTB rate of 8.0% versus 5.1% for those (*n* = 20,298) with a lifelong residence in non-mortgage discriminated neighborhoods; RR = 1.58 (1.18, 2.12).

**Conclusions:**

Urban African-American women’s early-life residence in predominately non-Hispanic White, mortgage discriminated neighborhoods is associated with an increased risk of PTB, particularly its’ early component, independent of adulthood risk status.

## Significance

What is Already Known on this Subject

What this Study Adds

Mortgage discrimination is a proxy of structural racism. A limited published literature highlights conflicting results with respect to the association of urban African-American women’s adulthood residence in mortgage discriminated neighborhoods and PTB rates.

Urban African-American women’s early-life residence in mortgage discriminated neighborhoods is a risk factor for PTB. This finding persists among those who also have an adulthood residence in non-mortgage discriminated neighborhoods. In stark contrast, this finding is absent among African-American women with early-life residence in non-mortgage discriminated neighborhoods and adulthood residence in mortgage discriminated areas.

In the United States, African-American women have a preterm birth (< 37 weeks, PTB) rate of 14.8% compared to 9.4% for non-Hispanic White women (Osterman et al., 2023). Moreover, the racial disparity is greatest for the early (< 34 weeks) component: 5.0% versus 2.3%, respectively (Osterman et al., 2023). Reflecting the strong relation of gestational age to first-year mortality, the disparate early PTB rates between racial groups is the primary determinant of excess number of deaths among African-American infants compared to White infants (Schempf et al., 2007; Riddell et al., 2017). The contribution of upstream factors linked to structural racism to African-American women’s birth outcome disadvantage is a burgeoning area of research (Collins & David, 2022; Matoba et al., 2019a, 2019b; Weiss et al., 2023).

Structural racism within the institution of mortgage lending has resulted in wealth inequities, disparate neighborhood investments, and interpersonal discrimination (Pearson et al., 2023). The association of historical redlining and current preterm birth risk is now well recognized in the context of persistent economic inequities and neighborhood infrastructure (Krieger et al., 2020; Nardone et al., 2020; Mehdipanah et al., 2023). Compounded by the consequences of historical redlining, contemporary mortgage discrimination practices continue to exclude minoritized populations access to high quality neighborhoods and their resources (Steil et al., 2018). However, the role of mortgage discrimination throughout the life-course in preterm birth risk is still incompletely understood (Mendez et al., 2014; Matoba et al., 2019a, 2019b).

A life-course conceptual model has been proposed to explain the discordant pregnancy outcome between US-born Black and non-Hispanic White women (Jones et al., 2019; Ben-Shlomo & Kuh, 2002; Lu & Halfon, 2003; Gluckman et al., 2008; Collins et al., 2011). In this model, the racial disparity reflects a greater prevalence of pre-pregnancy contextual risk factors and a lower prevalence of protective contextual factors among US-born Blacks compared to non-Hispanic Whites. The proposed mechanisms include in utero programming of reproductive potential and cumulative wear and tear, or weathering, and epigenetics. A prior study found that African-American women’s early-life residence in impoverished, urban neighborhoods was a risk factor for preterm birth independent of adulthood risk status, suggesting a contextual process (Collins et al., 2011). To our knowledge no study has investigated the impact of mortgage discrimination in preterm birth risk across the life-course.

Therefore, we designed a longitudinal transgenerational study to ascertain the extent to which urban African-American women’s early-life residence in mortgage discriminated neighborhoods is associated with PTB rates. We hypothesized that African-American women’s early-life residence in mortgage discriminated neighborhoods (compared to non-mortgage discriminated neighborhoods) is a contextual risk factor for PTB.

## Methods

### Chicago Transgenerational Birth-File (TGBF)

The Chicago Department of Public Health provided the 2005–2017 vital records of Chicago-born infants (*n* = 40,860) whose mothers (born 1989–1991) had been previously identified in the Illinois TGBF (David et al., 2010). Based on mother’s name, exact date of birth, and fuzzy matching to account for minor spelling errors, we linked 92% of infant (*n* = 37,403) vital records to their Chicago-born mothers previously captured in the Illinois TGBF.

In the 1920s, 77 community areas were created by demographers from the University of Chicago. They were intended to reflect community-formed neighborhoods and have remained consistent since their formation (Burgess & Newcomb, 1931). Based on maternal community area of residence at the time of delivery, 2007–2017 Home Mortgage Disclosure Act (HMDA) data and 2010–2017 US census income information were appended to the vital records of infants born 2005–2017. This information was used to assess maternal adulthood residential environment, particularly exposure to mortgage discrimination. Based on maternal grandmother’s place of residence at the time of delivery, the pooled 1990–1995 HMDA data and 1990 US census income information were appended to the vital records of mothers born 1989–1991 (Chicago Data Portal, 2003). This information was used to assess maternal early-life (fetal, infant, early childhood) residential environment, particularly exposure mortgage discrimination. All identifying information on individual infants and their mothers were removed after the linkage was completed. The institutional review board approved the research study.

### Study Sample

Maternal race and country of origin variables listed on the birth records were used to identify non-Hispanic African-American women. The study was restricted to singleton births of non-Hispanic African-American women aged 16 to 28 years with a lifelong residence in Chicago (*n* = 24,104).

### Loan Denial Odds Ratios

HMDA data was used to calculate loan denial odds ratios between African-American and non-Hispanic White applicants. The loan denial odds ratios for each community area of Chicago were estimated from empirical Bayes estimates of random intercept and slope mixed logistic regression model with applicant gross income, loan amount and applicant sex controlled for as fixed effects (Mendez et al., 2011). Additional variables such as credit, assets, and liabilities were not included as they are unavailable in the HMDA dataset.

Level 1 Equation:

$$\begin{aligned} \text{ln}\left[\frac{{p}_{ij}}{{(1-p)}_{ij}}\right]&={\beta}_{oj}+{\beta}_{1j}{\left(race\:of\:applicant\right)}_{ij}\\&\quad+{\beta}_{2j}{\left(gross\:annual\:income\right)}_{ij}\\&\quad+{\beta\:}_{3j}{\left(loan\:amount\right)}_{ij}\\&\quad+{\beta}_{4j}{\left(sex\:of\:applicant\right)}_{ij} \end{aligned}$$

Level 2 Equation:

$$\:{\beta\:}_{0j}\:=\:{\gamma\:}_{00}+{\mu\:}_{0j}$$

$$\:{\beta\:}_{1j}\:=\:{\gamma\:}_{10}+{\mu\:}_{1j}$$

$$\:{\beta\:}_{\rho\:j}\:=\:{\gamma\:}_{\rho\:0}+{\mu\:}_{\rho\:j}\left(for\:p>1\right)$$

$$\:\left[\frac{{\mu\:}_{0j}}{{\mu\:}_{1j}}\right]\:\sim\:N\left(\left[\frac{0}{0}\right],\left[\frac{{\tau\:}_{00}}{{\tau\:}_{10}{\tau\:}_{11}}\right]\right)$$

A loan denial odds ratio (OR) of 1.4 or greater was used to indicate community areas with mortgage discrimination (Matoba et al., 2019a, 2019b; Mendez et al., 2011). Community areas with less than 2 loans to either non-Hispanic White or African American applicants during 1990–1995 or 2007–2017 were excluded (*n* = 1 community area from 1990 to 1995). The presence of mortgage discrimination of African-American women’s community area of residence when she was born (1989–1991) and when her infant was born (2005–2017) were used to define early-life and adulthood exposure to mortgage discrimination, respectively. This variable was classified separately for each time period.

### Residential Segregation

The Index of Concentration at the Extremes (ICE) was used to measure residential segregation. ICE is a measure of spatial social polarization that quantifies the extremes of race/ethnicity, income, and combined race/ethnicity with income (Massey, 2001; Krieger et al., 2016). We used 1990 census demographic information to ascertain the ICE_race/ethnicity_ measure for community areas of African-American women at the time of their birth (1990–1995). The ICE_race/ethnicity_ was calculated using the following formula (Krieger et al., 2016):

$$IC{E_{race/ethnicity}}=\left( {Ai--Pi} \right)/Ti$$

A_i_ is the number of non-Hispanic Black populations per community area, P_i_ is the number of non-Hispanic White populations per community area, and T_i_ is the total population of the community area. The ICE_race/ethnicity_ value is a continuous measure from − 1 to + 1, in which − 1 indicates total concentration of non-Hispanic Black residents and + 1 indicates total concentration of non-Hispanic White residents. Limited by 1990 census data availability, ICE_income_ or ICE_combined race/ethnicity with income_ were unable to be calculated. Instead, median family income of community area was empirically divided into quartiles. We compared the distribution of racial make-up, racial segregation, and neighborhood income in early-life mortgage discriminated community areas and non-mortgage discriminated community areas.

### Statistical Analyses

The distribution of African-American women’s age, education, trimester of prenatal care initiation, parity, cigarette smoking was determined according to their early-life residence in mortgage discriminated versus non-mortgage discriminated neighborhoods, using chi-square tests to assess significant differences between groups.

We calculated rates of early (< 34 weeks), late (34–36 weeks), and total (< 37 weeks) PTB according to African-American women’s early-life exposure to mortgage discrimination. Multivariable log binomial regression models were constructed to better ascertain the independent association of African-American women’s early-life exposure to mortgage discrimination and PTB by gestational week categories. In all analyses, term (37 weeks and greater) rates were the referent outcome.

Lastly, we assessed the relationship between African American women’s change in exposure to neighborhood mortgage discrimination between early-life and adulthood compared to lifelong residence in non-mortgage discriminated neighborhoods. We used backwards elimination to assess for confounding by each maternal covariate, and the final adjusted model included trimester of entry into prenatal care and maternal smoking. No more than 8% of observations were missing in adjusted models due to incomplete covariate data. Data analysis was conducted in SAS version 9.4.

## Results

The mean ICE_race/ethnicity_ for mortgage discriminated neighborhoods equaled 0.78 (0.64–0.91) and the mean ICE_race/ethnicity_ for non-mortgage discriminated neighborhoods equaled − 0.18 (-0.36-0.00). Mortgage discriminated neighborhoods also had a greater proportion of high-income households, as defined by the fourth quartile median income households, compared to non-mortgage discriminated neighborhoods: 62% vs. 17%, *p* < 0.01.

There were no differences in the distribution of African-American women’s age, education, parity, trimester of prenatal care initiation, and cigarette smoking between those who had an early-life residence in mortgage discriminated versus non- mortgage discriminated neighborhoods (Table 1).

African-American women (*n* = 735) with early-life residence in mortgage discriminated neighborhoods had a PTB rate of 15.8% compared to 13.1% for those (*n* = 23,369) with early-life residence in non-mortgage discriminated neighborhoods; RR = 1.20 (1.01, 1.43). The RR of early and late PTB for African-American women with an early-life residence in mortgage discriminated (compared to non- mortgage discriminated) neighborhoods equaled 1.36 (1.04, 1.78) and 1.13 (0.69, 1.43), respectively.

In our multivariable log binomial regression models, the adjusted RR of early, late, and total PTB for African-American women with early-life residence in mortgage discriminated (compared to non- mortgage discriminated) neighborhoods equaled 1.60 (1.20, 2.14), 1.18 (0.92,1.53), and 1.31 (1.09,1.57), respectively (Table 2).

Table 3 shows PTB rates according to African-American women’s lifelong exposure to mortgage discrimination. The subgroup of African-American women (*n* = 536) with early-life residence in mortgage discriminated neighborhoods and adulthood residence in non-mortgage discriminated neighborhoods had an early PTB rate of 8.0% compared to 5.1% for those (*n* = 20,298) with a lifelong residence in non-mortgage discriminated neighborhoods; RR = 1.58 (1.18, 2.12). The subgroup of African-American women (*n* = 2,441) with an early-life residence in non-mortgage discriminated and adulthood residence in mortgage discriminated neighborhoods had an early PTB of 5.5%; RR = 1.09 (0.91, 1.30).

## Discussion

Our population-based investigation provides new information that African-American women’s early-life residence in mortgage discriminated (compared to non-mortgage discriminated) urban neighborhoods is a risk factor for early PTB independent of adulthood risk status. Interestingly, we found that a positive association of African-American women’s early-life residence in mortgage discriminated neighborhoods and early PTB rates persists among those with adulthood residence in non-mortgage discriminated neighborhoods.

Historical redlining was the racially based legalized system of mortgage discrimination implemented by the federal government following the passing of New Deal era laws and regulations. In the late 1930s, the Home Owners Loan Corporation created a series of residential security maps to classify neighborhoods by their perceived level of lending risk with redlining consisting of marking neighborhoods deemed hazardous, often poor-quality neighborhoods with majority minoritized populations, with a red line to indicate areas where banks should not invest (Richardson et al., 2020; Mendez et al., 2014; Bailey et al., 2021). These “risk based” lending maps perpetuated racial segregation, disinvestment, and economic inequities in neighborhoods predominately populated by low-income African-Americans (Greer, 2014).

In the vast majority of urban cities, historical redlining created enclaves of predominately impoverished, African-American neighborhoods (Bailey et al., 2021). Disturbingly, African-Americans continue to suffer mortgage discrimination from financial institutions (United States Department of Housing and Urban Development v. Associated Bank, N.A., 2015; National Fair Housing Alliance et al. v. Redfin Corporation, 2022). Studies have provided evidence that African-American women’s lifelong exposure to urban impoverishment is a contextual risk factor for adverse birth outcome (Collins et al., 2009). However, the present study illustrates that mortgage discrimination continues in predominantly high-income, non-Hispanic White neighborhoods, and is a lifelong risk factor for preterm birth in African-American women.

Few studies have investigated the impact of African-American women’s residence in mortgage discriminated neighborhoods on PTB rates, and the results are conflicting. Using a sample of Philadelphia vital records, Mendez et al. reported that mortgage discrimination was associated with a slightly decreased risk of PTB among African-American women (Mendez et al., 2014). In a larger study of Chicago vital records, Matoba et al. (2019a, 2019b) found that African-American women’s adulthood residence in mortgage discriminated neighborhoods was associated with a modest increased risk of PTB.

The present study suggests that African-American women’s differential exposure to early-life and adulthood neighborhood mortgage discrimination may be a contribution to the inconsistent findings. Our results show that African-American women’s early-life exposure to mortgage discrimination is associated with higher overall and early PTB rates even among the subgroup with adulthood residence in non-mortgage discriminated neighborhoods. Additionally, African-American women’s adulthood exposure to mortgage discrimination is not associated with PTB rates among the subgroup with an early-life residence in non-mortgage discriminated areas. Stratifying preterm birth into its early and late components provides additional importance on the epidemiology of birth outcomes as early prematurity is associated with greater morbidity and first-year mortality. Collectively, these findings suggest that African-American women’s early-life exposure to mortgage discrimination is a contextual risk factor for preterm birth below 34 weeks gestation.

**Fig. 1:**
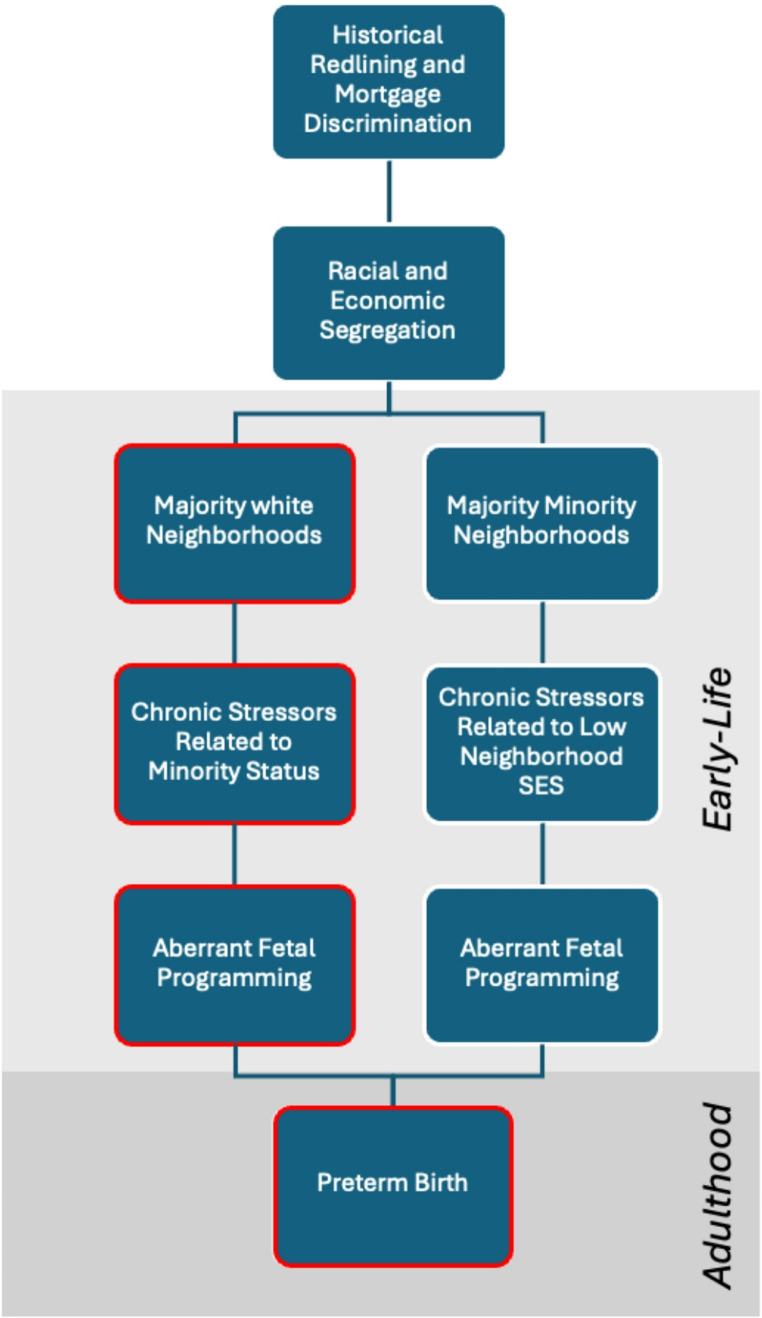
Conceptual model for early-life exposure to mortgage discrimination and preterm birth risk

We speculate that pregnant African-American women’s early-life residence in mortgage discriminated neighborhoods experience chronic stressors secondary to their minority status, and this phenomenon aberrantly programs their reproductive physiology via an epigenetic mechanism to deliver preterm infants in adulthood (Figure 1). A landmark study looking at the effects of the Dutch Famine on pregnant women provided epidemiological evidence of prenatal exposure of early-life stress on future metabolic dysregulation (Ravelli et al., 1998). Furthermore, adaptations to early-life psychosocial stress may result in dysregulated hypothalamo-pituitary-adrenocortical (HPA axis) and future preterm birth risk (Chen et al., 2024). A previous study by Gee (2008) also found that self-reported mental health of Chinese Americans was worse with greater reports of discrimination in redlined areas, characterized as areas with more Whites and fewer Chinese Americans (Gee, 2008). Placental transcriptome analyses have found significant differences in gene expression among early vs. late spontaneous preterm birth rates, supporting an epigenetic phenomenon of fetal programming especially on early preterm birth mediated by placental dysfunction. (Paquette et al., 2023). Neighborhoods with mortgage discrimination may represent a wider pattern of discrimination, highlighting its significance as a contextual risk factor. Future studies may benefit from including neighborhood level factors to further disentangle the effects of mortgage discrimination on birth outcomes. Greater attention to the impact of African-American women’s in utero, infancy, and early childhood residence in mortgage discriminated neighborhoods on their risk of adverse pregnancy outcome during adulthood is warranted.

Mortgage discrimination exemplifies structural racism, emphasizing the divergent impacts of socioeconomic opportunities on various levels of influence including both community and interpersonal discrimination (Alvidrez et al., 2019). The present study indicates that the occurrence of mortgage discrimination varies based on the racial makeup of a neighborhood, with African-Americans experiencing higher denial rates in predominantly White neighborhoods. We support public health efforts for fair housing policies designed to address segregation including low-income housing tax credits, economic mobility initiatives, and investment in minority neighborhoods. Redlining has evolved from historical legal policies to de facto discriminatory practices but continues to have significant public health consequences. A transgenerational multi-level approach is needed to confront deeply ingrained structural inequities and comprehensively tackle racial disparities in current birth outcomes.

To our knowledge, the Chicago TGBF is the only population-based dataset of infants and their mothers with appended life-course HMDA information; however, we acknowledged certain limitations. First, the dataset does not contain the vital records of births to Chicago-born African-American women who migrated out of Chicago. A prior study found that urban born African-American women’s geographic mobility to either contiguous or distant suburbs was associated with a lower risk of PTB (Collins et al., 2013). As such, our findings may not be generalizable beyond African-American women with a lifelong long urban residence. Second, we were only able to define African-American women’s exposure to mortgage discrimination at the time of her own birth and at time of delivery. We had no information on duration of early-life and adulthood exposure to mortgage discrimination or, the interval period. Notwithstanding, the assessment of residence in mortgage discriminated neighborhoods at two distinct time-periods is improvement over previous research with one measurement at the time of delivery. Thirdly, there were too few African-American women (*n* = 199) with early-life and adulthood residence in mortgage discriminated neighborhoods to calculate meaningful PTB rates. Fourthly, sample size considerations also restricted our ability to ascertain whether maternal birth weight modified the relation of early-life and adulthood residence in mortgage discriminated neighborhoods to PTB rates. Lastly, a priori we chose 1.4 as the cut-off value to define mortgage discriminated neighborhoods (Gee, 2008, Matoba et al., 2019a, 2019b; Mendez et al., 2011). Different cutoff points may have affected our findings (Edwards et al., 2024).

In summary, urban African-American women’s early-life exposure to mortgage discrimination is associated with an increased risk for PTB, particularly its’ early component.

**Table 1:** Distribution of African-American women’s selected characteristics according to early-life residential environment, Chicago vital records 1989–1991, 2005–2017

African-American women’s characteristics	Early-life residential environment	
	Non-mortgage discriminated (*n* = 23,369)	Mortgage discriminated (*n* = 735)
Age (years)	%	%
< 20	35.5	36.3
20–24	47.1	45.6
25+	17.4	18.0
Education (years)		
< 12	34.2	34.7
12	34.2	33.5
> 12	31.6	31.8
Parity		
Primiparous	52.7	53.6
1 Previous live birth	27.8	27.7
2 Previous live births	12.3	11.7
3 Or more previous live births	7.2	7.0
Prenatal care initiation		
None	4.3	3.2
First trimester	65.6	66.4
Second or third trimester	30.1	30.4
Cigarette smoker		
No	95.0	94.8
Yes	5.3	5.2

**Table 2:** African-American women’s early (< 34 weeks), late (34–36 weeks), and total (< 37 weeks) preterm birth (PTB) rates according to early-life residential environment, Chicago vital records 1989–1991, 2005–2017

Early life residential environment	PTB rate (per 100)	aRR (95% CI)
Early PTB (< 34 weeks)		
Non-mortgage discriminated	5.2	Ref
Mortgage discriminated	6.9	1.60 (1.20, 2.14)
Late PTB (34–36 weeks)		
Non-mortgage discriminated	8.0	Ref
Mortgage discriminated	8.8	1.18 (0.92,1.53)
Total PTB (< 37 weeks)		
Non-mortgage discriminated	13.1	Ref
Mortgage discriminated	15.8	1.31 (1.09,1.57)

*Controlling for prenatal care and smoking

**Table 3:** African-American women’s early (< 34 weeks), late (34–36 weeks), and total (< 37 weeks) preterm birth (PTB) rates according to early-life and adulthood residential environment, Chicago vital records 1989–1991, 2005–2017

Early-life and adulthood residential environments	Early PTB rate (per 100)	RR (95% CI)	Late PTB rate (per 100)	RR (95% CI)	Total PTB rate (per 100)	RR (95% CI)
Early-life and adulthood non-mortgage discriminated (*n* = 20,928)	5.1	Ref	7.8	Ref	13.0	Ref
Early-life and adulthood mortgage discriminated (*n* = 199)	4.0	0.80 (0.40, 1.57)	9.1	1.14 (0.73, 1.77)	13.1	1.01 (0.70, 1.45)
Early-life mortgage discriminated and adulthood non-mortgage discriminated (*n* = 536)	8.0	1.58 (1.18, 2.12)	8.8	1.15 (0.88, 1.52)	16.8	1.30 (1.07, 1.57)
Early-life non-mortgage discriminated and adulthood mortgage discriminated (*n* = 2,441)	5.5	1.09 (0.91, 1.30)	9.1	1.16 (1.01, 1.33)	14.5	1.12 (1.01, 1.24)
